# Emergency Medicine Resident Rotations Abroad: Current Status and Next Steps

**DOI:** 10.5811/westjem.2015.11.28159

**Published:** 2016-01-12

**Authors:** Stephen C. Morris, Erika D. Schroeder

**Affiliations:** * University of Washington School of Medicine, Division of Emergency Medicine, Seattle, Washington; † North Sound Emergency Medicine, Everett, Washington

## Abstract

**Introduction:**

International rotations for residents are increasingly popular, but there is a dearth of evidence to demonstrate that these rotations are safe and that residents have appropriate training and support to conduct them.

**Methods:**

A survey was sent to all U.S. emergency medicine (EM) residencies with publicly available e-mail addresses. The survey documents and examines the training and support that emergency medicine residents are offered for international rotations and the frequency of adverse safety events.

**Results:**

72.5% of program director responded that their residents are participating in rotations abroad. However, only 15.4% of programs reported offering training specific to working abroad. The results point to an increased need for specific training and insurance coverage.

**Conclusion:**

Oversight of international rotations should be improved to guarantee safety and education benefit.

## INTRODUCTION

International rotations for emergency medicine (EM) residents are becoming increasingly popular, but little is known about institutional support for this trend, specifically, educational supervision, safety activities, and insurance coverage related to these rotations. This research sought to determine how many EM programs in the United States were sending their residents abroad and the safety, training and insurance status of the residents, as well as any adverse events that occurred.

## BACKGROUND

Over the last three decades the amount of funding for and projects in global health have significantly increased, as has awareness of global health issues within the mainstream U.S. medical system. At the same time, there has been an exponential surge in interest and involvement in international rotations among medical students and residents in the U.S.^1^,^2^ Despite increased opportunities and funding, there is unmet demand from individuals who would like to work abroad.

By 2004, 22% of U.S. medical students had participated in an international rotation.^3^ Although there are many competing demands on a trainee’s time during residency, many U.S. programs allow participation in international rotations. As a specialty, EM engages in all aspects of the medical spectrum; thus, there are few clinical specialties as well suited to the global health clinical environment. Additionally, given the dynamic, high-paced, and unpredictable nature of EM, there are likely common characteristics that make global health interesting to EM providers. Several studies have shown that a significant percentage of EM residents participate in these rotations and that, at this point, there is no standardization of training for trainees who work abroad.^4^,^5^

## METHODS

This study was given a waiver by Institutional Review Board (IRB) of the University of Washington.

We conducted an online search of the 165 U.S.-based EM residency programs during the summer of 2013. The name of the program director or assistant director and their email was obtained from the residency website. If no contact information was available, the residency was not included in the survey.

The authors created a nine-question survey that could be completed in 2–3 minutes. The study was opened on September 13, 2013, and closed on June 16, 2014.

In the fall of 2013, the survey was sent to the 134 EM programs that had publicly available email addresses. Individuals who did not complete the survey were sent a series of reminder emails requesting participation or removal from the survey. The researchers made a single phone call to those who had not completed the survey requesting their participation towards the end of the survey period. The results were permanently uncoupled from the respondent’s email as per the requirement of University of Washington’s IRB waiver criteria.

The first three questions identified the number of residents in the program, the length of the training program, and whether any of the residents participated in international rotations. Questions 4 and 5 inquired whether the program had dedicated global health faculty and an established international site for resident rotations. Question 6 asked if residents were given any training prior to participating in an international rotation and question 7 addressed the presence of liability coverage of the residents working abroad. Question 8 attempted to ascertain how rotation sites were assessed for safety and question 9 allowed respondents to chronicle safety events that had occurred to their residents during the preceding five years.

## RESULTS

A total of 91 of 134 residency program or assistant program directors answered the survey for a response rate of 67.9%. The respondents represent 55% of all EM programs. Of the 91 responders, 66 (72.5%) responded that their residents participated in rotations abroad as part of their training program. An average 10.4% and 8.86% of residents in three- and four-year programs, respectively, were reported to have participated in a rotation longer than two weeks during the 12 months prior to the survey.

Of all respondents, only 17.6% responded that they had three or more faculty whose primary areas of academic interest is global health; 38.5% reported their program had an “established relationship (defined as frequent educational contact, one or more ongoing projects, and faculty or resident exchange) with an emergency department or training facility outside of the U.S.”

An estimate of the nature of the residents’ international work was given by 35 respondents. When asked to estimate the type of work performed abroad by category, results demonstrated clinical work 38%, EM development 18%, and public health work 12%. Project work that was less frequently conducted included observational and research, 9% each, and humanitarian response and disaster response, 7% each. Unfortunately and interestingly, many respondents did not complete this question.

Only 15.4% of programs reported offering any special training prior to allowing their residents to work abroad. Of the small percentage of respondents who reported conducting some training, the themes were public health skills, personal health and safety, tropical medicine and ethical considerations. Liability insurance covered the residents’ work abroad at 47% of the responders’ institutions, while 31% were not covered and 22% were unsure of their institutional policies.

Of the 65 responders who answered if their program sites where evaluated for safety by faculty, 29 (45%) said “yes” while 36 (55%) said “no” or were unsure. Several responders cited some other mechanism for safety evaluation including using local staff and online screening of political security. Very few security events and no deaths were reported by the respondents. Of note, 12% of responders reported major illness or political instability affecting their residents and 2% reported assault or an incident requiring evacuation. Key findings are summarized in Figure.

## LIMITATIONS

This study used a publicly generated list of residency directors in order to be eligible for IRB waiver and repeated contacts. This resulted in an incomplete survey of EM residency programs; however, our data on the number of residents participating is similar to prior studies. The study relied on the memory of the respondents resulting in potential recall bias. In a few cases both the residency director and assistant residency director may have responded to the survey resulting in redundant submissions. In addition, our study over represented four-year programs. Nationally four-year residencies represent 24.6% of all EM residencies while we had 37.5% of our responses from four-year residencies.

## DISCUSSION

Our study revealed both positive and negative characteristics of the current policies regarding international rotations in EM. Of greatest importance to the authors, there are a large number of residents going abroad every year and overall the level of insecurity they are experiencing from health or safety risks is low. However, pre-travel training, vetting of work sites, presence of global health faculty and ensured liability are all lower than expected. Clinical work, EM development and public health projects dominated the nature of the work, while research, humanitarian and disaster work are at surprisingly low levels.

Perhaps the most important outcome of this study is that it highlights the need for a uniform and comprehensive national education program for EM residents doing international rotations. A global health component of resident education, pre-departure safety training and use of attending physician-vetted sites to enhance resident safety and the quality of the educational experience should be considered minimum requirements for any program that allows residents to participate in rotations abroad. Pre-departure appropriate course work in working in austere environments, cultural sensitivity, tropical disease and public health should be considered. Use of mentorship should also be encouraged to maximize the resident’s experience and provide additional outlets for accessing faculty.

## CONCLUSION

Our survey pointed out several important issues regarding international rotations for EM. First, as with other specialties, international rotations are common and heterogeneous with regard to activities conducted and supervision. Additionally, there is a low level of pre-rotation training, insurance coverage and site safety evaluation. Finally though safety incidents are rare, several serious events including major illness, assault and injury were reported. This study highlights the need for greater supervision, training and support of EM residents conducting rotations abroad.

## Figures and Tables

**Figure:**
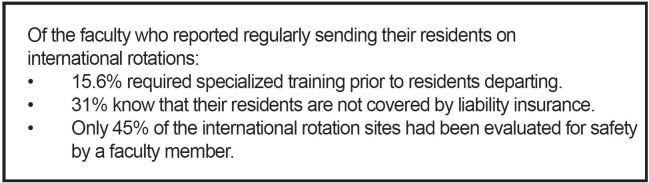
Summary of results.
